# Alert Override Patterns With a Medication Clinical Decision Support System in an Academic Emergency Department: Retrospective Descriptive Study

**DOI:** 10.2196/23351

**Published:** 2020-11-04

**Authors:** Junsang Yoo, Jeonghoon Lee, Poong-Lyul Rhee, Dong Kyung Chang, Mira Kang, Jong Soo Choi, David W Bates, Won Chul Cha

**Affiliations:** 1 Institution of Healthcare Resource School of Nursing Sahmyook University Seoul Republic of Korea; 2 Samsung Advanced Institute for Health Sciences & Technology (SAIHST) Department of Digital Health Sungkyunkwan University Seoul Republic of Korea; 3 Department of Gastroenterology Samsung Medical Center Sungkyunkwan University School of Medicine Seoul Republic of Korea; 4 Health Information and Strategy Center Samsung Medical Center Seoul Republic of Korea; 5 Center for Health Promotion Samsung Medical Center Sungkyunkwan University School of Medicine Seoul Republic of Korea; 6 Division of General Internal Meidicine and Primary Care Brigham and Women's Hospital Boston, MA United States; 7 Department of Emergency Medicine Samsung Medical Center Sungkyunkwan University School of Medicine Seoul Republic of Korea

**Keywords:** medical order entry systems, decision support systems, clinical, alert fatigue, health personnel, clinical decision support system, alert, emergency department, medication

## Abstract

**Background:**

Physicians’ alert overriding behavior is considered to be the most important factor leading to failure of computerized provider order entry (CPOE) combined with a clinical decision support system (CDSS) in achieving its potential adverse drug events prevention effect. Previous studies on this subject have focused on specific diseases or alert types for well-defined targets and particular settings. The emergency department is an optimal environment to examine physicians’ alert overriding behaviors from a broad perspective because patients have a wider range of severity, and many receive interdisciplinary care in this environment. However, less than one-tenth of related studies have targeted this physician behavior in an emergency department setting.

**Objective:**

The aim of this study was to describe alert override patterns with a commercial medication CDSS in an academic emergency department.

**Methods:**

This study was conducted at a tertiary urban academic hospital in the emergency department with an annual census of 80,000 visits. We analyzed data on the patients who visited the emergency department for 18 months and the medical staff who treated them, including the prescription and CPOE alert log. We also performed descriptive analysis and logistic regression for assessing the risk factors for alert overrides.

**Results:**

During the study period, 611 physicians cared for 71,546 patients with 101,186 visits. The emergency department physicians encountered 13.75 alerts during every 100 orders entered. Of the total 102,887 alerts, almost two-thirds (65,616, 63.77%) were overridden. Univariate and multivariate logistic regression analyses identified 21 statistically significant risk factors for emergency department physicians’ alert override behavior.

**Conclusions:**

In this retrospective study, we described the alert override patterns with a medication CDSS in an academic emergency department. We found relatively low overrides and assessed their contributing factors, including physicians’ designation and specialty, patients’ severity and chief complaints, and alert and medication type.

## Introduction

An emergency department (ED) is a challenging environment in which multiple interventions are delivered within a short period [1]. The severity of the patients’ conditions demands that providers often order medications and tests simultaneously, which could contribute to a higher rate of medical errors [2-4]. Physicians working in an ED must often make decisions in the context of uncertainty due to the pace of the environment and resource limitations [5]. Specifically, the concept of physicians working in an ED is not limited to emergency medicine specialists, but rather covers various medical department physicians who treat patients in the geographical area of the ED.

Computerized provider order entry (CPOE) combined with a clinical decision support system (CDSS) was introduced to reduce preventable adverse drug events [6]. This system was expected to improve physicians’ prescribing patterns by supporting their decision-making process in a variety of ways. However, previous studies have revealed that physicians’ override rates on CDSS alerts are high [7-10], raising concerns about the effectiveness of CDSSs in many implementations [11-13].

Many factors, including physician and patient characteristics, environmental factors, and factors associated with the system itself, affect physicians’ alert override patterns in multifactorial ways with probable interactions among them [14-16]. Additionally, many previous studies regarding physicians’ alert override patterns have focused on specific diseases or alert types for well-defined targets as well as particular settings [10,17,18]. Thus, it is not clear how these results will generalize to patients at large or in settings such as the ED. Moreover, alert-related fatigue and physician burnout are very frequent among ED physicians, and also appear to be associated with worse performance of a CDSS [19-21].

Based on this background, the aim of this study was to describe and assess alert override patterns with a medication CDSS in a large academic ED.

## Methods

### Study Setting

**Figure 1:**
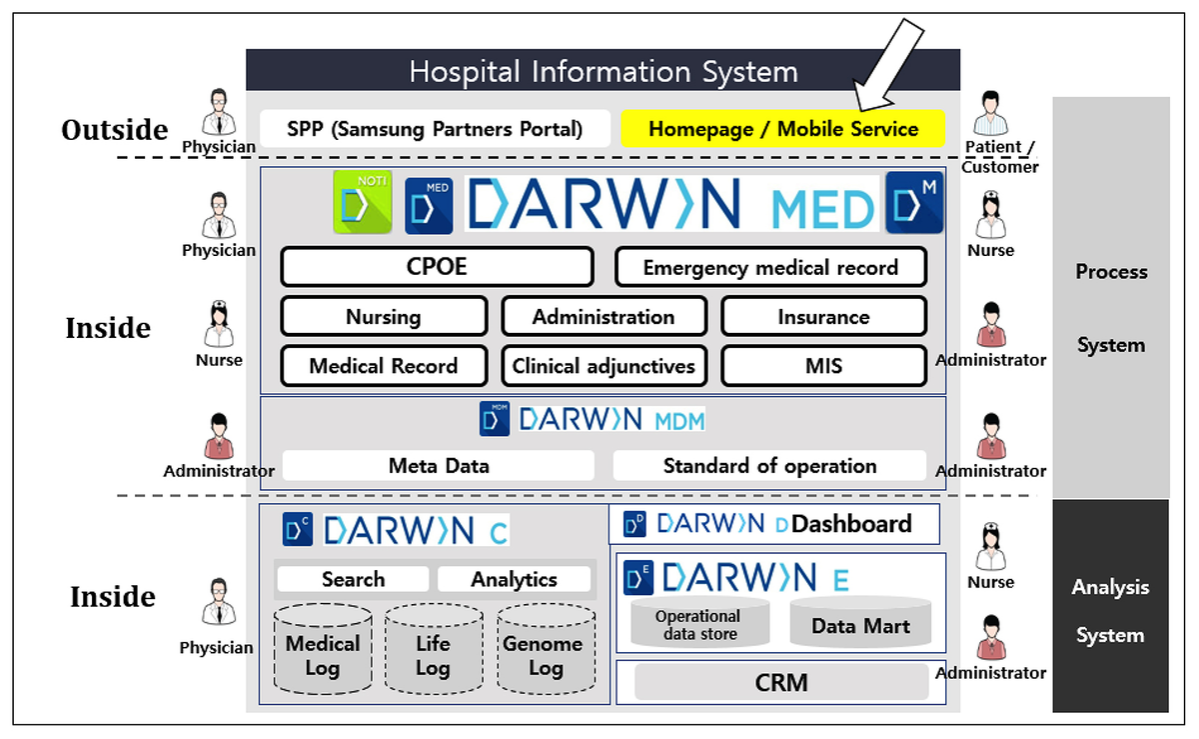
Overall schematic description of the hospital information system architecture at the Samsung Medical Center. DARWIN: Data Analytics and Research Window for Integrated Knowledge; CPOE: computerized physician order entry; MIS: management information system; MDM: master data management; CRM: customer relationship management. Reproduced with permission from Jung et al [22].

This study was conducted at an ED with an annual visit volume of 80,000 patients. The hospital is an academic institute with 2000 inpatient beds. The institution has utilized a home-grown electronic health record (EHR) system since 2003, which was replaced by a next-generation EHR system named Data Analytics and Research Window for Integrated Knowledge (DARWIN) in 2016. DARWIN is an all-in-one home-grown EHR that includes CPOE, nursing, pharmacy, billing, research support, and a patient portal (Figure 1) [22]. The institution’s ethics committee approved this study (Institutional Review Board File No. 2019-05-038).

### Minimally Interruptive CDSS

**Figure 2:**
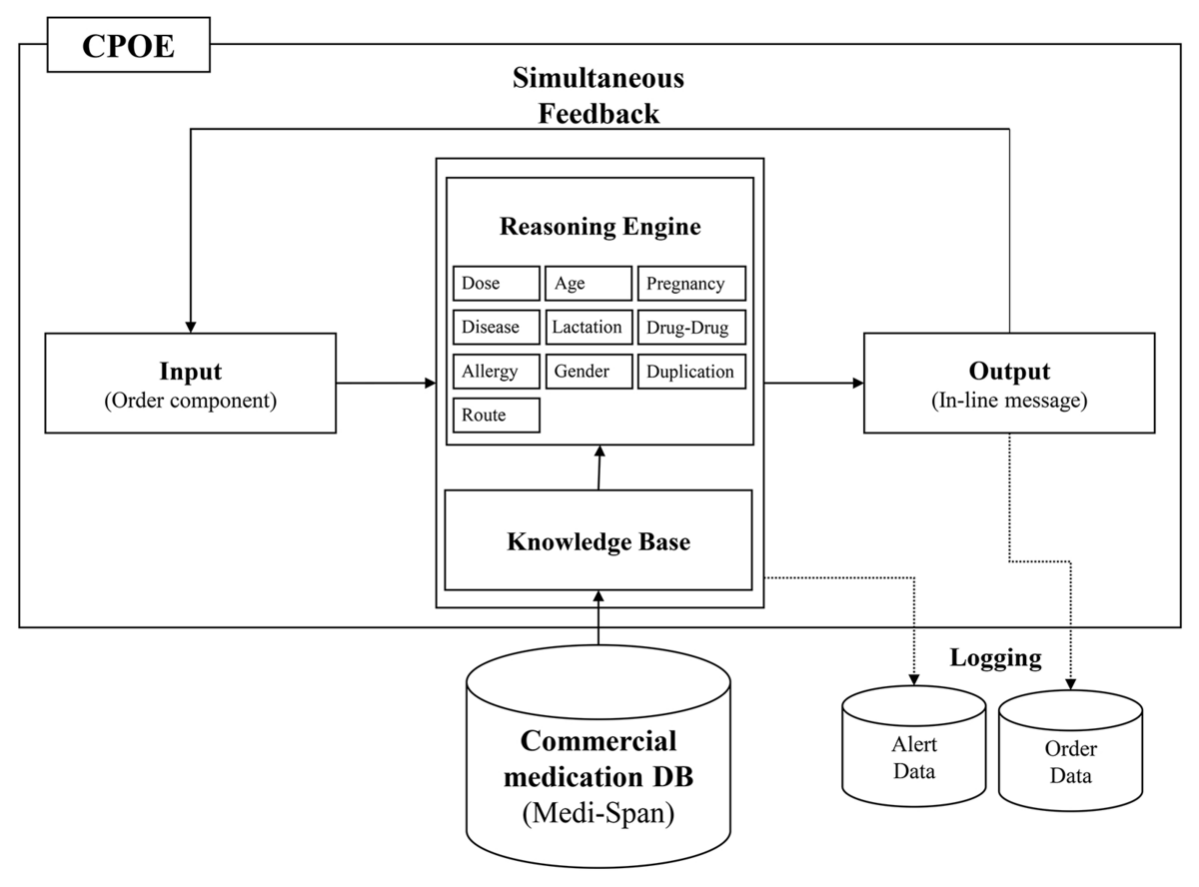
System architecture of the computerized provider order entry (CPOE). DB: database.

When developing DARWIN’s CDSS, a minimally interruptive medication CDSS was introduced. This CDSS is mainly designed for physicians and utilizes only medication-specific information so that, for instance, there is no interference with laboratory data. This database is supplied from Medi-Span (Wolters Kluwer Health, Philadelphia, PA, USA) and is updated monthly (Figure 2).

**Figure 3:**
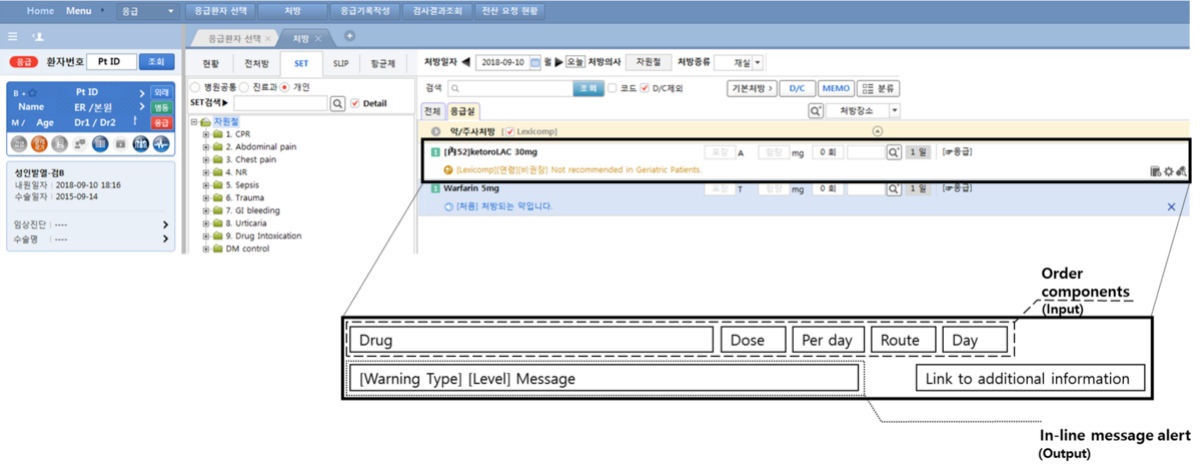
Screenshot of the computerized provider order entry system and features of its interface (zoomed out).

The user interface was designed to minimize interruption in physician prescription workflow. First, the rules engine operates simultaneously with the physician’s entry of each order component such as a drug name, dose, and route. Second, its feedback appears as an in-line message so that physicians are not interrupted during order processing (Figure 3). The CDSS operates with the following areas of medication: age, allergy, disease, duplication, gender, lactation, pregnancy, route, drug-drug interaction, and dosage.

### Study Subjects

**Figure 4:**
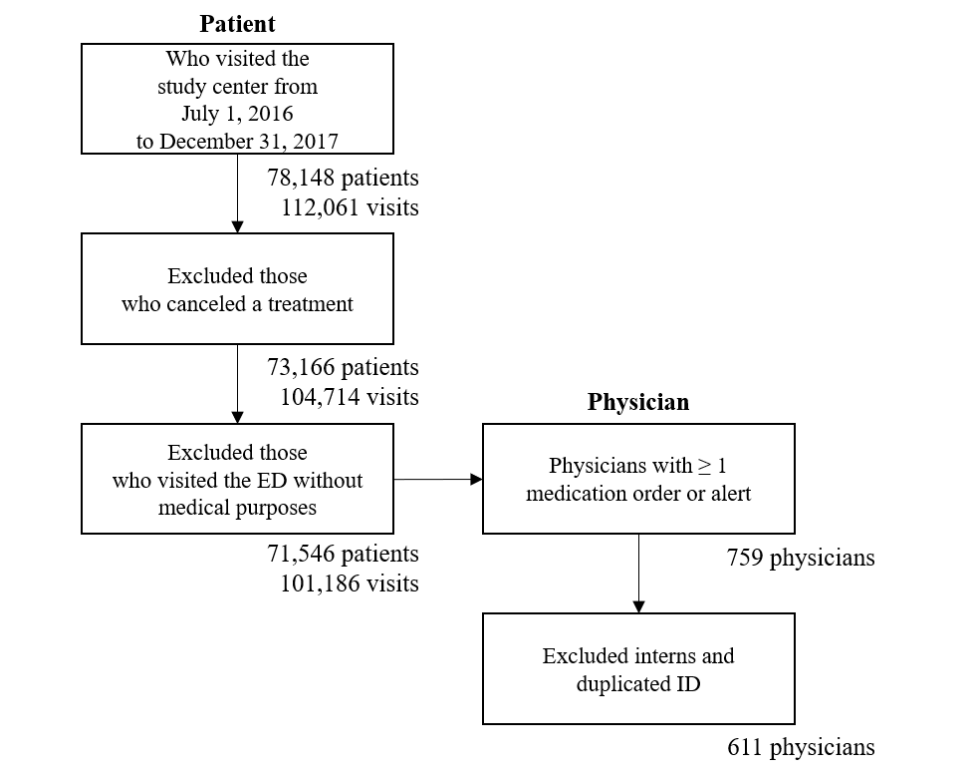
Flow diagram of the eligibility and selection process for study inclusion. ED: emergency department; ID: identification.

The inclusion criteria for this study were that patients had to have visited the ED between July 1, 2016 and December 31, 2017. Patients were excluded if they visited the ED but left without being examined by physicians or if they visited the ED without a medical purpose. The eligibility and selection process is presented in Figure 4. As we aimed to extensively investigate alert override patterns in an ED, the term “physician in ED” includes physicians from various medical departments, including the ED, pediatrics, internal medicine, and plastic surgery, among others.

### Data Extraction and Preparation

Clinical data were extracted from the clinical data warehouse of the study site. We collected the following data: patient information (deidentified patient identifier, date of birth, gender, chief complaint, visit time, type of disposition, length of ED stay, severity level, and International Classification of Diseases-10 code), alert information (medication code based on the generic product identifier, alert firing time), order information (medication code, order time), and physician information (physician identifier, department, and career status). The severity score was measured by the Korean Triage and Acuity Scale (KTAS), which has been widely used by triage nurses in Korean EDs [23]. The KTAS was developed based on the Canadian Triage Scale to assess ED visiting patients’ acuity and severity. Patients with a score corresponding to level 1 have the highest acuity and severity, whereas level 5 indicates the lowest acuity and severity.

### Override Determining Algorithm

An override determining algorithm was developed for assessing the outcome measures. The algorithm was based on a rule-based, alert type–specific logic, newly generated for this study for validation (Table 1).

**Table 1:** Description of the alert overrides determining logic.

Type of alert	Override determining logic
Age, allergy, disease, duplication, gender, lactation, pregnancy, route	If a physician completed the order without alert adjustment
Dose	If a physician did not adjust the dose-related order components such as prescription day or daily dosage
Drug-drug interaction	If a physician ordered both medications indicated in a drug-drug interaction alert

For the chart review, we selected a sample of 20 alerts among each type of alert that was performed for both overridden and nonoverridden orders. In the first round of the review process, two clinicians independently reviewed the sample alerts and then evaluated the interrater reliability using the Cohen κ statistic. In the second round, both clinicians worked together to resolve any case of disagreement. The two clinicians who reviewed the log data consisted of a doctor and nurse who have worked at the ED of the study site for over 4 years. Accuracy of the override determining algorithm was assessed using the reviewed data as the gold standard.

### Data Analysis

We conducted a descriptive analysis of patients, physicians, alert characteristics, and the alert firing and override rates. We used a logistic regression model for assessing the risk of the alert override using scalable medical variables such as physician factors (physicians’ specialties and designation), patient factors (severity scores and chief complaints), and alert factors (types of alerts and medication categories of alerts). The statistical significance level was set at *P*<.05. The variable with the smallest difference between the overall mean override rate and overrode rate of each variable (eg, resident) within each group (eg, physicians’ designation) was selected as the reference variable of the logistic regression. We employed R (version 3.6.0) software for the analysis.

## Results

### Interrater Reliability of the Override Determining Algorithm

In the first round of the review process conducted by two independent clinicians, Cohen κ was 0.82 (95% CI 0.74-0.90). All discrepancies were resolved in the second round of the review process. The accuracy of the override determining algorithm was 0.95.

### Basic Characteristics

During the study period, 611 physicians took care of 71,546 patients with 101,186 visits. General characteristics of the physicians and patients are described in Table 2 and Table 3, respectively. The physicians prescribed 748,339 medication orders and 102,887 (13.75%) alerts were fired.

**Table 2:** Physician characteristics (N=611).

Characteristic		n (%)
**Designation**		
	Resident	357 (58.3)
	Fellow	154 (25.2)
	Faculty	100 (16.4)
**Specialty**		
	Emergency Medicine	41 (6.7)
	General Internal Medicine	60 (9.8)
	Gastroenterology	39 (6.4)
	Cardiology	17 (2.8)
	Pulmonary Medicine	15 (2.5)
	Nephrology	13 (2.1)
	Hematology & Oncology	10 (1.6)
	Endocrinology & Metabolism	8 (1.3)
	Infectious Disease	7 (1.2)
	Allergic Medicine	2 (0.3)
	Rheumatology	2 (0.3)
	General Surgery	58 (9.5)
	Gynecology & Obstetrics	39 (6.4)
	Thoracic surgery	26 (4.3)
	Orthopedic Surgery	19 (3.1)
	Neurosurgery	15 (2.5)
	Urology	15 (2.5)
	Plastic Surgery	11 (1.8)
	Pediatrics	53 (8.7)
	Family Medicine	24 (3.9)
	Ophthalmology	24 (3.9)
	Otolaryngology	24 (3.9)
	Neurology	20 (3.3)
	Radiology	16 (2.6)
	Psychiatry	14 (2.3)
	Anesthesiology & Pain Medicine	10 (1.6)
	Dermatology	9 (1.5)
	Critical Care Medicine	7 (1.2)
	Rehabilitation	7 (1.2)
	Dentistry	5 (0.8)
	Radiation Oncology	1 (0.2)

**Table 3:** Patient characteristics (N=101,186 visits).

Characteristic		Value
Age (years), mean (SD)		44.50 (25.68)
Male, n (%)		51,221 (50.62)
**Severity score, n (%)**		
	1 (Highest severity)	1122 (1.11)
	2	6331 (6.25)
	3	38,456 (38.01)
	4	46,696 (46.15)
	5 (Lowest severity)	8581 (8.48)
**Chief complaint, n (%)**		
	Fever	15,080 (14.90)
	Abdominal Pain	14,285 (14.12)
	Dyspnea	6920 (6.84)
	Minor Complaint	6536 (6.46)
	Dizziness	4920 (4.86)
	Headache	3643 (3.60)
	Laceration	2366 (2.34)
	Skin Rash	2323 (2.30)
	Head Trauma	2259 (2.23)
	Pain (Lower Extremity)	2011 (1.99)
	Chest Pain (Suspected Cardiogenic Pain)	1859 (1.84)
	Injury (Upper Extremity)	1820 (1.80)
	Injury (Lower Extremity)	1734 (1.71)
	Pain (Upper Extremity)	1639 (1.62)
	Limb Weakness	1488 (1.47)
	Inter-Hospital Transfer	1434 (1.42)
	Altered Mentality	1393 (1.38)
	Back Pain	1317 (1.3)
	Coughing and Stuffy Nose	1197 (1.18)
	Palpitation and Irregular Heart Rate	1196 (1.18)
	Hematochezia/Melena	1169 (1.16)
	Seizure	1154 (1.14)
	Nausea/Vomiting	1106 (1.09)
	General Weakness	1037 (1.02)
	Injury (Facial)	994 (0.98)
	Chest Pain (Noncardiogenic)	860 (0.85)
	Hematuria	854 (0.84)
	Other	18,592 (18.37)

### Override Patterns

Of the total 102,887 alerts, 65,616 (63.77%) alerts were overridden. We then analyzed the effects of physician-related factors, patient-related factors, and alert-related factors that could affect the physicians’ alert override behavior (Table 4).

**Table 4:** The risk of alert overrides according to various factors.

Factor				Frequency of alert (n)		Alert override rate, n (%)		Univariate logistic regression odds ratio (95% CI)		Multivariate logistic regression odds ratio (95% CI)
**Physician-related factors**										
	**Physicians’ Designation**									
		Resident	93,022		59,678 (64.15)		1 [Reference]		1 [Reference]	
		Fellow	8174		4993 (61.08)		0.88 (0.84-0.92)		0.9 (0.86-0.94)	
		Faculty	1691		945 (55.88)		0.71 (0.64-0.78)		0.73 (0.66-0.81)	
	**Physicians’ Specialty**									
		Emergency Department	50,812		32,542 (64.04)		1 [Reference]		1 [Reference]	
		Internal Medicine	17,476		10,623 (60.79)		0.87 (0.84-0.9)		1.03 (0.99-1.07)	
		Surgical Department	8737		5501 (62.96)		0.95 (0.91-1)		0.90 (0.85-0.94)	
		Other Department	25,862		16,950 (65.54)		1.07 (1.03-1.1)		1.10 (1.06-1.14)	
**Patient-related factors**										
	**Patients’ severity score**									
		1 (Highest Severity)	1597		912 (57.11)		0.80 (0.73-0.89)		0.82 (0.74-0.91)	
		2	8985		5421 (60.33)		0.92 (0.88-0.96)		0.89 (0.85-0.94)	
		3	45,759		28,536 (62.36)		1 [Reference]		1 [Reference]	
		4	41,171		27,059 (65.72)		1.16 (1.13-1.19)		1.07 (1.04-1.10)	
		5 (Lowest Severity)	5375		3688 (68.61)		1.32 (1.24-1.40)		1.23 (1.15-1.32)	
	**Patients’ chief complaints**									
		Fever	26,334		16,769 (63.68)		1 [Reference]		1 [Reference]	
		Abdominal Pain	11,300		6525 (57.74)		0.78 (0.75-0.82)		0.95 (0.91-1.00)	
		Altered Mentality	3220		2080 (64.6)		1.04 (0.96-1.12)		1.49 (1.37-1.62)	
		Back Pain	1684		1079 (64.07)		1.02 (0.92-1.13)		1.04 (0.94-1.16)	
		Chest Pain (Non-Cardiogenic)	680		388 (57.06)		0.76 (0.65-0.88)		0.84 (0.72-0.98)	
		Chest Pain (Suspected Cardiogenic Pain)	1946		1216 (62.49)		0.95 (0.86-1.05)		1.22 (1.09-1.37)	
		Coughing and Stuffy Nose	1195		769 (64.35)		1.03 (0.91-1.16)		1.04 (0.92-1.18)	
		Dizziness	3128		2098 (67.07)		1.16 (1.07-1.26)		1.65 (1.52-1.79)	
		Dyspnea	8432		4894 (58.04)		0.79 (0.75-0.83)		0.93 (0.88-0.98)	
		General Weakness	1419		909 (64.06)		1.02 (0.91-1.14)		1.28 (1.15-1.44)	
		Head Trauma	1327		985 (74.23)		1.64 (1.45-1.86)		1.64 (1.45-1.87)	
		Headache	4165		2723 (65.38)		1.08 (1.01-1.15)		1.08 (1.01-1.16)	
		Hematochezia/Melena	3236		2209 (68.26)		1.23 (1.13-1.33)		2 (1.84-2.18)	
		Hematuria	561		379 (67.56)		1.19 (1.00-1.42)		1.25 (1.05-1.50)	
		Injury (Facial)	556		446 (80.22)		2.31 (1.88-2.87)		2.18 (1.76-2.70)	
		Injury (Lower Extremity)	1335		905 (67.79)		1.2 (1.07-1.35)		1.29 (1.14-1.46)	
		Injury (Upper Extremity)	1103		817 (74.07)		1.63 (1.42-1.87)		1.56 (1.36-1.80)	
		Inter-hospital Transfer	1354		855 (63.15)		0.98 (0.87-1.10)		1.10 (0.97-1.24)	
		Laceration	1834		1585 (86.42)		3.63 (3.18-4.17)		3.43 (2.98-3.95)	
		Limb Weakness	1173		689 (58.74)		0.81 (0.72-0.91)		1.01 (0.89-1.14)	
		Minor Complaint	5082		3408 (67.06)		1.16 (1.09-1.24)		1.18 (1.10-1.27)	
		Nausea/Vomiting	921		455 (49.4)		0.56 (0.49-0.64)		0.7 (0.61-0.80)	
		Pain (Lower Extremity)	1800		1111 (61.72)		0.92 (0.83-1.02)		0.97 (0.88-1.08)	
		Pain (Upper Extremity)	1043		731 (70.09)		1.34 (1.17-1.53)		1.3 (1.13-1.49)	
		Palpitation and Irregular Heart Rate	693		404 (58.3)		0.8 (0.68-0.93)		0.98 (0.84-1.15)	
		Seizure	1993		1179 (59.16)		0.83 (0.75-0.91)		0.93 (0.84-1.02)	
		Skin Rash	1776		1141 (64.25)		1.02 (0.93-1.13)		1.18 (1.06-1.32)	
		Others	13,597		8867 (65.21)		1.07 (1.02-1.12)		1.29 (1.23-1.35)	
**A** **lert-related factors**										
	**Type of alert**									
		Duplication	1414		911 (64.43)		1 [Reference]		1 [Reference]	
		Age	17,949		11,035 (61.48)		0.88 (0.79-0.99)		0.8 (0.71-0.90)	
		Allergy	1583		822 (51.93)		0.6 (0.51-0.69)		0.54 (0.46-0.62)	
		Disease	4041		2479 (61.35)		0.88 (0.77-0.99)		0.93 (0.82-1.06)	
		Dose	67,212		43,873 (65.28)		1.04 (0.93-1.16)		0.99 (0.88-1.11)	
		Drug-Drug Interaction	1399		926 (66.19)		1.08 (0.93-1.26)		1.07 (0.91-1.25)	
		Gender	381		183 (48.03)		0.51 (0.41-0.64)		0.43 (0.33-0.56)	
		Lactation	18		13 (72.22)		1.44 (0.54-4.50)		1.33 (0.50-4.18)	
		Pregnancy	8651		5199 (60.10)		0.83 (0.74-0.93)		0.72 (0.64-0.81)	
		Route	239		175 (73.22)		1.51 (1.12-2.06)		1.19 (0.88-1.64)	
	**Medication categories (based on generic product ID)**									
		Neuromuscular Drugs	1511		961 (63.60)		1 [Reference]		1 [Reference]	
		Analgesics and Anesthetics	36,685		24,808 (67.62)		1.20 (1.07-1.33)		1.23 (1.10-1.38)	
		Anti-Infective Agents	18,308		12,419 (67.83)		1.21 (1.08-1.35)		1.08 (0.96-1.21)	
		Antineoplastic	326		198 (60.74)		0.89 (0.69-1.13)		0.94 (0.73-1.22)	
		Biologicals	192		138 (71.88)		1.46 (1.06-2.05)		1.09 (0.78-1.53)	
		Cardiovascular Agents	5866		3637 (62)		0.93 (0.83-1.05)		0.95 (0.84-1.07)	
		Central Nervous System Drugs	5434		3066 (56.42)		0.74 (0.66-0.83)		0.67 (0.59-0.76)	
		Endocrine and Metabolic Drugs	3374		2009 (59.54)		0.84 (0.74-0.95)		0.84 (0.74-0.96)	
		Gastrointestinal Agents	15,589		8619 (55.29)		0.71 (0.63-0.79)		0.6 (0.53-0.67)	
		Genitourinary Agents	343		211 (61.52)		0.91 (0.72-1.17)		1.42 (1.07-1.90)	
		Hematological Agents	4938		3210 (65.01)		1.06 (0.94-1.20)		1.04 (0.91-1.18)	
		Miscellaneous Products	1168		711 (60.87)		0.89 (0.76-1.04)		0.92 (0.78-1.09)	
		Nutritional Product	1843		1214 (65.87)		1.1 (0.96-1.27)		1.09 (0.94-1.26)	
		Respiratory Agents	6778		3994 (58.93)		0.82 (0.73-0.92)		0.85 (0.75-0.96)	
		Topical Products	532		421 (79.14)		2.17 (1.72-2.75)		1.62 (1.27-2.07)	

In terms of physician-related factors, the resident group had a higher override rate than both the fellow and faculty groups. The top three physicians’ specialty departments that generated the most alerts in the ED were the emergency medicine, pediatrics, and general internal medicine departments. Physicians working in the ED were found to override over 64% of the total alerts received. In terms of patient factors, the alert override rate tended to decrease with the increase in severity. Additionally, the alert override rate also showed wide variation according to the patients’ chief complaints. The override rate tended to be higher in patients with trauma such as laceration and injuries than in patients with other chief complaints.

Two-thirds of the total alerts were dose alerts, and 65.3% of these were overridden. ED physicians overrode approximately half of the gender- and allergy-type alerts. Regarding the medication group, alerts for gastrointestinal agents, central nervous system drugs, respiratory agents, and endocrine and metabolic drugs showed the lowest override rates. Among the variables used in multivariate logistic regression models, the following variables emerged as statistically significant risk factors: miscellaneous department group in physician’s specialty; patients with lower and lowest severity; the presence of dizziness, head trauma, headache, hematochezia, melena, hematuria, facial injuries, lower extremity injuries, upper extremity injuries, laceration, minor complaints, and upper extremity pain; drug-drug interaction, lactation, and the route; and medication categories of analgesics and anesthetics, anti-infective agents, and topical products (Table 4).

## Discussion

### Principal Results

In this study, we examined the alert override patterns in an ED with a CDSS that was designed in a minimally interruptive way. Approximately two-thirds of the alerts were overridden, which is a lower rate than reported in many previous related studies. We assessed many covariates, including both physician-related and patient-related factors, as well as alert-related factors. These results could be used for optimizing and maximizing the effectiveness of the CDSS.

### Physicians’ Specialty, Patients’ Severity, and Alert Override Rate

Many studies examining alert override rates have not examined the physicians’ designations as an input variable or did not find it to have a significant effect [14,24]. Despite lack of evidence, it is generally considered that experienced physicians do not need alerts because “they already know” [25], and even if they receive them, they may be more likely to override them. In contrast to this expectation, we found that the alert override rate was the highest among residents, followed by fellows and senior faculty members (Table 4). This finding establishes that even an experienced physician still requires assistances from a CDSS.

We also demonstrated that physicians override more alerts in patients with complaints of lower severity. The override rate for patients with the highest severity level was 57.1% and that for patients with the lowest severity level was 63.5%. The override rate decreased significantly as the patients’ severity increased (Table 4). To date, relatively little attention has been paid to patients’ severity as a covariate affecting physicians’ alert overriding behavior. Further investigation is needed to confirm this finding.

### Comparison With Prior Work

#### Low Override Rate

One of the major findings in this study is the relatively low override rate. The low override rate was observed consistently across the three factors (physician, patient, and alert), implying that a systematic influence exists aside from those discussed above. One potential explanation could be related to the phenomenon that has been termed the “cloud of context.” Coiera et al [26] proposed that “variations in the workflow, patient population and morbidity, resources, pre-existing infrastructure, and the education and experience of both clinical staff and patients” function as a “cloud of context” that affect how physicians respond to a CDSS. Given that most of the existing CDSS studies were conducted in inpatient and outpatient settings in the United States, these contextual factors may have influenced the lower override rate observed in this study. Previous studies have reported that override rates increased from 72.8% to 93% [7-10,24]. In comparison, a Korean study reported a rate of 71.7% [14], which is relatively lower than that reported for the United States. However, there are no other ED-based studies that we are aware of for direct comparison; thus, the conclusion warrants further investigation.

Another possible explanation is the system design. It is well known that utilizing a human factors design reduces the error rate and that an interactive design can reduce alert fatigue [15,27]. We leveraged several strategies to ensure that the system is integrated into the clinical workflow by designing a noninterruptive system. For example, the concept of timely alerts was implemented more seamlessly by designing a system that can generate an alert whenever a new order component is entered. Although our research scope did not include direct measurements and analysis of usability, we believe that integrating the system into the clinical workflow contributes to the override rate for ED physicians. We also believe that the underlying knowledge used was relatively robust.

#### Importance of the ED Environment in Alert Override–Related Research

In this study, we assessed the alert overriding patterns of ED physicians in routine care in the ED. An ED is an optimal environment to examine physicians’ alert overriding behaviors from a broad perspective because patients have a wider range of severity than those in other departments, and many receive interdisciplinary care in this environment. This viewpoint is important because the CDSS does not influence physicians concerning a single type of alert but rather acts as a bundle of user interfaces.

To the best of our knowledge, this is the first study to analyze physicians’ CDSS usage patterns in an ED comprehensively. In a recent review, less than one-tenth of the studies were found to target physicians’ behavior in the ED [15]. Some research studies only targeted specific alert types such as drug-drug interaction or opioid alerts, or only a specific group such as pediatric patients [10,17,18]. In this study, the analysis was performed on the whole system rather than focusing on particular types of alerts, physicians, or patients. The effect of a CDSS may be critical in this complex environment, and the results of this study may play an essential role in establishing CDSSs in EDs or in cases in which a multidisciplinary approach is needed.

### Limitations

First, this study was performed in a single ED with a homegrown EHR. The ED part of an academic referral center receives patients with conditions of high severity, and the majority of physicians are trainees of its residency program. Furthermore, the EHR, DARWIN, has only been implemented in a few hospitals in Korea to date, which should be considered while generalizing our results.

Second, the alert override was the only outcome measured. The outcome of the alert override or the reason for override was not recorded in the database. Thus, it was not possible to determine the clinical appropriateness of the alerts or overrides, or the consequences for patients. Therefore, there is a need for a follow-up interview study or an institution-level investigation of adverse drug events.

Third, the effect of the minimally interrupted CDSS was not demonstrated in this ED. For clarity, a comparative study is required. Such a comparison would require a control group with more interruptive alerts on similar targets. The nature of the CDSS makes it difficult to perform a randomized controlled trial.

Finally, we did not scale all potential factors related to alert overrides in previous studies, such as alert fatigue, alert severity, and workload. In particular, to estimate the size of the effects of alert fatigue, there is a need for further observational studies using devices such as eye trackers that can quantitatively measure whether the physician paid attention to the CPOE alert.

### Conclusions

In this retrospective study, we described alert override patterns with a medication CDSS in an academic ED. We found a relatively low rate of overrides and also assessed the influence of multiple contributing factors on these rates. This study could aid CDSS implementers by providing knowledge regarding physicians’ alert overriding behaviors as well as empirical evidence that contradicts conventional notions.

## Abbreviations

CDSS: computerized decision support system
CPOE: computerized provider order entry
DARWIN: Data Analytics and Research Window for Integrated Knowledge
ED: emergency department
EHR: electronic health record
KTAS: Korean Triage and Acuity Scale
